# Predictors of readmission in a medical department of a tertiary university hospital in the Philippines

**DOI:** 10.1186/s12913-023-09608-z

**Published:** 2023-06-12

**Authors:** Janika Adrienne L. Balane, Celina Daia DG. Yap, Cary Amiel G. Villanueva, Lia Aileen M. Palileo-Villanueva, Diana R. Tamondong-Lachica

**Affiliations:** 1 Department of Medicine, University of the Philippines- Philippine General Hospital, Manila, Philippines; 2 University of the Philippines-College of Medicine, University of the Philippines Manila, Manila, Philippines; 3 Department of Medicine, Division of Adult Medicine, University of the Philippines- Philippine General Hospital, Manila, Philippines

**Keywords:** Hospital readmission, Predictors, Health service, Quality improvement

## Abstract

**Background:**

Identifying factors that increase the risk for hospital readmission helps in determining potential targets for quality improvement efforts. The main objective of this study was to examine factors that predict increased risk of hospital readmission within 30 days of hospital discharge of patients under the General Medicine service of a tertiary government hospital in Manila, Philippines.

**Methods:**

We performed a retrospective cohort study which included service patients 19 years old and above readmitted within 30 days following discharge. A total of 324 hospital readmissions within 30 days of discharge from January 1 to December 31, 2019 were reviewed. We estimated the rate of 30-day readmission and identified factors associated with preventable readmissions using multivariable logistic regression.

**Results:**

Of the 4,010 hospitalizations under General Medicine service in 2019, 602 (18%) were readmissions within 30 days of discharge, majority of which were related to the index admission (90%) and unplanned (68%). Predictors of preventable readmission were emergency readmission (OR 3.37, 95% CI 1.72 to 6.60), having five to ten medications at discharge (OR 1.78, 95% CI 1.10 to 2.87), and presence of nosocomial infection (OR 1.86, 95% CI 1.09 to 3.17). The most frequent reason for readmission among preventable ones is health-care related infection (42.9%).

**Conclusions:**

We identified factors which increased the likelihood of preventable readmissions such as type of readmission, number of medications per day, and presence of nosocomial infections. We propose that these issues be addressed to improve healthcare delivery and reduce readmission-related expenditures. Further studies should be pursued to identify impactful evidence-based practices.

**Supplementary Information:**

The online version contains supplementary material available at 10.1186/s12913-023-09608-z.

## Background

The appropriate use and allocation of hospital resources are growing concerns in several countries because healthcare service generates the largest expenditure. In connection with this, unnecessary and inappropriate use of these resources is a prominent issue. Thus, careful assessment of health service represents a useful strategy to improve the quality of healthcare delivered to patients.

Hospital readmission is regarded as an indicator of the quality of health services and remains a significant contributor to health care costs [1]. Quality issues are now a growing concern in hospital services [2]. Although not all readmissions are preventable, unplanned readmissions could be prevented by addressing risk factors that reflect poor health service or ineffective transition to home. Moreover, patient demographics and specific disease characteristics are associated with increased risk of hospital readmission [3–5].

Hospital readmission is typically defined as patient admission to a hospital within a certain period after being discharged from the same hospital [6]. A 30-day time window is commonly used to assess readmissions [1–5, 7, 8]. Worldwide, readmission rates (RaR) vary from 10% to 25% [3]. Many factors have been highlighted as contributors to hospital readmission. Common causes include frailty, progression of chronic disease, number of comorbidities, system-related issues such as lack of access to information or counseling, premature discharge [3] failure to fully treat the diagnosed condition or incomplete investigation, poor discharge planning, hospital acquired infection and inadequate therapy [9], Female gender, residency >35 km from the hospital, longer than average and very short hospital stays; and higher comorbidity scores were also associated with higher risk of rehospitalization [2]. Rehospitalization means poorer quality of life and a reflection of inadequate delivery of care. There is a paucity of exploration among these factors in the Philippines hence the need to further investigate which of them are relevant in our country. To our knowledge, there was only one published study that assessed hospital readmission in the Philippines.

The perception of frequent readmissions by hospital staff at a government-funded academic medical center led to this study. This tertiary hospital has a high referral rate which can allow gathering of comparative data for this study. These readmissions remain a significant contributor of healthcare costs especially in public institutions. Thus, identifying factors that increase risk for hospital readmissions can help in determining potential targets for quality improvement efforts.

Identifying factors that increase the risk for hospital readmission helps determine potential targets for quality improvement efforts. Thus, this study aimed to examine factors associated with 30-day readmission of adult patients admitted in a medical department of a tertiary university hospital in Metro Manila, Philippines. In doing so, this study may help improve delivery of quality health care and serve as precedent to future studies on quality improvement in our country.

## Methods

### Study design and setting

We performed a retrospective cohort study of all 30-day readmissions in the General Medicine services of Philippine General Hospital (PGH) for the period of January 1, 2019-December 31, 2019.

The Philippine General Hospital, a tertiary government hospital in Manila, Philippines, is designated as the National University Hospital and caters to the diverse and complex health needs of Filipinos. Many patients come to the hospital as both out-patient and in-patient to seek consultation and admission, sometimes leading to a surge from all over the country. In PGH, there has been a high number of admissions seen leading to full capacity. Those admissions include not just new cases but also several readmissions accounting for a high number of patients.

### Study population

We included all patients 19 years old and above readmitted within 30 days following discharge under the General Medicine service for the index admission covering the period of 1 January 2019 to 31 December 2019. All readmissions for elective procedures such as chemotherapy, diagnostic procedures, surgery and delivery were included as well as multiple readmissions of one patient within the given period. Patients admitted in another service who are transferred to General Medicine as primary service during admission were included. The following patients were excluded: those who were previously discharged against medical advice and who absconded. Planned and unplanned readmissions were taken into account to oversee distribution of admissions as these may play a role in allocation of limited resources (e.g. bed capacity, number of wards).

We operationally defined index admission as any admission that could be followed by a readmission. A readmission is when a patient who has been discharged from PGH is admitted again within 30 days of discharge. It could be a planned readmission for elective surgery, chemotherapy, diagnostic procedures and delivery or unplanned readmission due to emergency purposes (i.e., medical, surgical emergencies, trauma, delivery).

### Study variables

We preselected predictor variables to be collected following the previously identified factors affecting readmission based on a review of the literature: age grouped into four categories (19–29, 30–49, 50–65, > 65) [2], sex, marital status, age- adjusted Charlson Comorbidity Index (ACCI) to quantify the overall burden of comorbid conditions [10], place of residence (urban and rural), distance of residence from PGH which reflects ease of access to health care, employment status, social classification which is a surrogate for socioeconomic status as determined by the medical social worker, type of index hospital admission and readmission (emergency or scheduled), day of index admission and discharge ( weekday or weekend) and length of hospital stay during index admission categorized into intervals: < 1, 1–4, 4–7, 7–14 and > 14 days [2].

Moreover, nosocomial infection as a reason for readmission was also reviewed as this was one of the biggest contributors for a 30-day readmission [3]. Nosocomial infection was defined as hospital-acquired infections which occur in a patient >48 hours after the index admission or within 90 days from hospitalization. Lastly, the number of medications and doses per day were also reviewed [11].

### Outcomes

Study outcomes include the factors associated with readmission in the Department of Medicine of Philippine General Hospital and 30-day readmission rate. A 30-day readmission rate index (RaR) index is a common measure of readmission rate and is extensively used in health services research concerning the hospitalization process ^2^. We defined the readmission rate (RaR) index as [11]:

$$\mathrm{RaR }= (\mathrm{Rn }+\mathrm{ n}+30) / (\mathrm{Hn }-\mathrm{ Rn})$$

where “Rn” denotes readmission in the year 2019, “n+30” refers to the first 30 days of the year 2020, which is the period when readmissions for hospitalizations from the last month of 2019 took place and Hn refers to the total number of admissions in the year 2019.

### Study procedures

A list of all admitted patients under the General Medicine service from January 2019 to December 2019 was retrieved from the hospital admitting section. Readmission within 30 days following discharge amongst these patients was reviewed using the computerized registry of admissions and discharges in the hospital.

Using electronic and physical charts from the Medical Records Section, records of all eligible patients readmitted within 30 days were identified by a trained research assistant. The data that were gathered cover index admissions from January 1, 2019 until December 31, 2019 and since we used a 30-day period for defining readmission, we also checked hospitalizations from January 2020 to identify hospital readmissions for which the index hospitalization took place in December 2019.

The readmissions were then classified into four categories by the 2 researchers independently (JALB and CDDY): (A) Planned and related to the index admission, (B) Planned and unrelated to the index admission, (C) Unplanned and related to the index admission, and (D) Unplanned and unrelated to the index. Planned and unplanned readmissions were classified based on their definition mentioned above (see study population). Two of the investigators classified the factors as preventable and non-preventable.

Preventable factors include: complication of surgical procedure except healthcare-related infection, procedure not performed during previous admission, surgical treatment that did not reach the proposed objective, lack of diagnosis during previous admission, health-care related infection, suboptimal medical treatment, unstable clinical condition at discharge from previous admission, inadequate use of drugs (eg. includes inadequate dosage and interactions), complication of diagnostic test, nonadherence to treatment allegedly due to lack of information, diagnostic and/or therapeutic problems that should have been treated by primary care services (eg. ambulatory, GP) and lack of appropriate alternative centers for delivering the care required (eg.palliative). Nonpreventable factors include unavoidable recurrence of disease, unavoidable progression of disease, process not related to previous episodes, planned readmission, nonadherence to therapeutic recommendations attributable to the patient including financial status, living conditions (lack of necessary resources) and patient’s preference and beliefs, adverse reaction to drugs, acute exacerbation of concomitant process and uncontrollable social problem [12].

Disagreements regarding the reason for admission and its relationship to the index admission were resolved by discussion among the two investigators (JALB and CDDY).

### Data management and analysis

Data gathered were encoded into a Microsoft Excel spreadsheet by a trained research assistant. In compliance with the Philippine Data Privacy Act of 2012, all patients were given code numbers instead of using their full names or initials in the study database.

Categorical variables were presented as frequencies and proportions. Univariable logistic regression was employed to determine the crude association of each variable to having preventable readmissions. Multivariable logistic regression with backward elimination at an alpha of 0.05 was then utilized to determine which of the variables were associated with having preventable readmissions. Odds ratios (ORs) with their corresponding 95% confidence intervals (CI) were obtained. All analyses were done in STATA 16.1/IC.

## Results

Among 4,010 hospital episodes reviewed, there were 324 patients with a total of 602 readmissions within 30 days. Thus, the General Medicine service had a RaR of 18%.

About half of readmitted patients were female (52%), married (53%) and had age-adjusted CCIs of 2–3 (45%) (Table 1). Most readmitted patients came from the lowest socioeconomic classification (98%) and lived in urban areas (86%).

**Table 1:** Characteristics of readmitted patients (*N* = 324)

Variable	N (%)
**Age (in years)**	
19–29	83 (25.6%)
30–49	112 (34.6%)
50–65	99 (30.6%)
> 65	30 (9.3%)
**Sex**	
Female	168 (51.9%)
Male	156 (48.2%)
**Marital Status**	
Single	131 (40.4%)
Married	171 (52.8%)
Widow/Separated	22 (6.8%)
**Age-Adjusted Charlson Comorbidity Index**	
0–1	36 (11.1%)
2–3	147 (45.4%)
4–5	77 (23.8%)
> 6	64 (19.8%)
**Residence**	
Rural	46 (14.2%)
Urban	278 (85.8%)
**Distance (in kilometers)**	
< 15	108 (33.3%)
16–35	99 (30.6%)
> 35	117 (36.1%)
**Employment**	
Unemployed	283 (87.4%)
Employed	41 (12.7%)
**MSS Classification**	
A	0
B	0
C	8 (2.5%)
D	316 (97.5%)
**Type of Index Admission**	
Emergency	265 (81.8%)
Scheduled	59 (18.2%)
**Day of index admission**	
Weekday	254 (78.4%)
Weekend	70 (21.6%)
**Day of discharge**	
Weekday	234 (72.2%)
Weekend	90 (27.8%)
**Length of hospital stay during index admission (days)**	
< 1	0
1–4	70 (21.6%)
4–7	74 (22.9%)
7–14	102 (31.5%)
> 14	78 (24.1%)
**Nosocomial infection**	
Yes	85 (26.2%)
No	239 (73.8%)
**Number of medications per day**	
< 5	158 (48.8%)
5–15	164 (50.6%)
> 15	2 (0.6%)
**Number of doses per day**	
< 10	207 (63.9%)
10–26	114 (35.2%)
> 26	3 (0.9%)

More than 90% of readmissions were related to the index admission with 68.2% being unplanned and 22.2% being planned (Supplementary table 1). Readmissions unrelated to the index admission were mostly unplanned 8.6%.

The type of index admission and of readmission, length of stay (LOS), having nosocomial infection, the number of medications and doses per day, and the readmission category were found to be associated with preventable admissions among patients (*p* < 0.05) (Table 2). Patients were twice as likely to have preventable readmissions if they had an emergency index admission (OR 2.36 [95% CI 1.27 to 4.41]) or had a nosocomial infection (OR 2.53 [95% CI 1.52 to 4.20]). Patients who received 5–15 medications per day were also two times more likely to be readmitted with a preventable reason compared to those with less than 5 medications per day (OR 2.30 [95% CI 1.47 to 3.62]).

**Table 2:** Simple logistic regression for unadjusted association of variables with having a preventable readmission

Variable	Preventable readmission/ total %	Crude odds ratio (95% Confidence Interval)	*p*-value
**Age (in years)**			
19–29	34 /83 (41)	Ref	-
30–49	48/112 (42.9)	1.08 (0.61–1.92)	0.791
50–65	42/99 (42.4)	1.06 (0.59–1.92)	0.842
> 65	16/30 (53.3)	1.65 (0.71–3.82)	0.244
**Sex**			
Female	74/168 (44.1)	Ref	-
Male	66/156 (42.3)	0.93 (0.60–1.45)	0.752
**Marital Status**			
Single	52/131 (39.7)	Ref	-
Married	77/171 (45)	1.24 (0.78–1.97)	0.353
Widow/Separated	11/22 (50)	1.52 (0.61–3.76)	0.366
**Age-Adjusted Charlson Comorbidity Index**			
0–1	16/36 (44.4)	Ref	-
2–3	55/147 (37.4)	0.75 (0.36–1.56)	0.439
4–5	35/77 (45.5)	1.04 (0.46–2.31)	0.920
> 6	34/64 (53.1)	1.42 (0.62–3.22)	0.405
**Residence**			
Rural	16/46 (34.8)	Ref	-
Urban	124/278 (44.6)	1.51 (0.79–2.90)	0.215
**Distance (in kilometers)**			
< 15	42/108 (38.9)	Ref	-
16–35	47/99 (47.5)	1.42 (0.82–2.47)	0.213
> 35	51/117 (43.6)	1.21 (0.71–2.07)	0.475
**Employment**			
Employed	13/41 (31.7)	Ref	-
Unemployed	127/283 (44.9)	1.75 (0.87–3.52)	0.115
**MSS Classification**			
C	5/8 (62.5)	Ref	-
D	135/316 (42.7)	0.45 (0.11–1.91)	0.277
**Type of Readmission**			
Scheduled	13/72 (18.1)	Ref	-
Emergency	127/252 (50.4)	4.61 (2.41–8.82)	< 0.001
**Type of Index Admission**			
Scheduled	16/59 (27.1)	Ref	-
Emergency	124/265 (46.8)	2.36 (1.27–4.41)	0.007
**Day of index admission**			
Weekday	143/254 (56.3)	Ref	-
Weekend	41/70 (58.6)	1.10 (0.64–1.88)	0.734
**Day of discharge**			
Weekday	133/234 (56.8)	Ref	-
Weekend	51/90 (56.7)	0.99 (0.61–1.62)	0.978
**Length of hospital stay during index admission (days)**			
1–4	19/70 (27.1)	Ref	-
4–7	37/74 (50.0)	2.68 (1.34–5.39)	0.005
7–14	50/102 (49.0)	2.58 (1.34–4.97)	0.005
> 14	34/78 (43.6)	2.07 (1.04–4.14)	0.039
**Nosocomial infection**			
No	89/239 (37.2)	Ref	-
Yes	51/85 (60.0)	2.53 (1.52–4.20)	< 0.001
**Number of medications per day**			
< 5	52/158 (32.9)	Ref	
5–15	87/164 (53.1)	2.30 (1.47–3.62)	< 0.001
> 15	1/2 (50.0)	2.03 (0.13–33.24)	0.617
**Number of doses per day**			
< 10	76/207 (36.7)	Ref	-
10–26	63/114 (55.3)	2.13 (1.34–3.39)	0.001
> 26	1/3 (33.3)	0.86 (0.08–9.66)	0.904
**Readmission category**			
Planned related to index admission	14/72 (19.4)	Ref	-
Planned unrelated to index admission	0/3	-	-
Unplanned related to index admission	111/221 (50.2)	4.18 (2.20–7.93)	< 0.001
Unplanned unrelated to index admission	15/28 (53.6)	4.78 (1.86–12.29)	0.001

In a multivariable model adjusted for nosocomial infection and number of medications (5–10 vs < 5 per day), an emergency readmission was three times more likely to be preventable compared to a scheduled readmission (OR 3.37 [95% CI 1.72 to 6.60]).(Supplementary table 2).

The most frequent reason for readmission among preventable readmissions is health-care related infection (42.9%) whereas planned readmissions are most frequent among non-preventable admissions. Other usual reasons for preventable readmissions include nonadherence to treatment allegedly due to lack of information (20%), procedures not performed during index admission (12.9%) as well as lack of diagnosis during previous admission (9.3%). For non-preventable admissions, other usual reasons include unavoidable progression of disease such as malignancies (24.5%), processes not related to previous admission (15.2%), as well as acute exacerbation of concomitant processes (8.2%). ( Supplementary table 3).

## Discussion

The interest to know the possible reasons for readmissions led to this study. There was only one published study that explored readmissions in the country. The overall readmission rate of the patients in a prospective cohort study conducted in the same institution was 17.9%. They concluded that the primary indication for readmission was the recurrence of the index condition and emphasized the role of healthcare providers as an important factor for timely follow up in the outpatient department [13]. In this study, we investigated factors contributing to 30-day readmissions. The number of 30-day readmission in 2019 was 602, translating to a RaR value of 18% which falls between the worldwide readmission rate ranging from 10–25%.

As to the reasons for readmission, most patients had non preventable reasons for readmission (57%). However, a significant number of 140 (43%) had preventable reasons for admission. These results were similar to the study by Dreyer et.al. which showed higher non-preventable readmissions with 35% being potentially preventable [3]. In another study by Bianco et.al., 43.7% of hospital readmissions were judged as potentially preventable [12]. In our observation, there seems to be a slightly higher proportion of non-preventable readmissions compared with preventable ones. Since the percentage for preventable readmissions is significant, more efforts must be made in addressing them.

The most common cause of readmission in our study was the presence of health-care related infections (42.9%). Hospital readmission and healthcare associated infections are common healthcare dilemmas with significant clinical and financial implications to both patients and hospital institutions. In the study by Dreyer, et.al., presence of nosocomial infections was one of the biggest contributors for a 30-day readmission [3]. This finding may be used to further assess health outcomes after discharge and employ better infection control and prevention strategies in the hospital.

The next most common cause is nonadherence to treatment allegedly due to lack of information (20%). In a study by Kryz et.al., only a few number of readmissions were due to patient’s noncompliance to therapeutic recommendations after index admissions [2]. This is in contrast with our findings wherein possible factors contributing to non-compliance such as cost of medications and ineffective discharge planning methods involving both patients and caregivers are still issues that need to be addressed.

Procedures not performed during the previous admission (12.9%) ranked as the third common cause. In the study by Bianco et.al., they cited procedures not performed in the first hospitalization (24%) to be one of the major reasons for preventable readmissions [12]. This suggests that the hospital should place a greater focus on diagnostics procedures which could be part of quality improvement projects. Furthermore, the lack of appropriate alternative centers for delivering the care required (eg. palliative) was also a reason for readmission. In the Philippines, we only have limited hospice care facilities especially for cancer patients.

Of the non preventable, the most common was planned readmission (39.1%), this includes admission for blood transfusion, chemotherapy, post chemotherapy monitoring, iron chelation and biopsy. Unavoidable progression of disease (24.5%) was also a common cause of non-preventable readmissions. This is similar to the study by Bianco et.al., a frequent reason for non-preventable readmission is planned readmission (25.6%), followed by unavoidable recurrence of disease (21.7%), and acute exacerbation of disease (20.2%) [12]. This tertiary hospital in our study is the only hospital in the region with the highest referral rate in all medical specialties. It treats the most complicated and difficult cases which are more susceptible to progression of disease hence readmission.

In one retrospective cross-sectional study conducted in Singapore that evaluated drug-related problem (DRP) readmissions, the study showed that noncompliance (5.6%) was the most common iatrogenic cause of readmission. Data on readmissions were treated as DRP related only when they were explicitly stated on the chart which was based on physician judgement. The study also showed that readmission frequencies increase with the number of doses (18.0 ± 8.0) and medications per day (10.0 ± 4.4) [11]. Our study shows that a number of medications per day increases the risk of preventable readmissions. Hence, healthcare providers can decrease frequency of readmission by providing simplified regimen with the use of longer acting alternatives or fixed dose combinations, avoidance of polypharmacy especially in the elderly and proper care planning prior to discharge.

Several limitations were encountered in this study. First, our information was limited since the data came from previous medical records and were retrospective in nature. There are a multitude of other patient characteristics we have not obtained (such as education, functional capacity measures, as well as measurement of pre-morbid health status) which could further help us determine potentially avoidable factors. We were not able to include data from all readmissions; rather we just focused on our sample size which did not accurately reflect the rest of the patient's characteristics. There is also instrument bias that may have limited our ability to detect potential factors for risk of readmission and account for confounding factors as well. In the future, we recommend more comprehensive investigations to these factors as well as collections using prospective study designs. Second, there is a subjective nature regarding our assessment of the reasons for readmission because we relied on physician perceptions based on what they documented. Hence, a more objective way should be done to identify other reasons for admission and results should be analyzed carefully. We recommend future studies which can address these validation markers to produce more objective reasons. Lastly, the readmissions identified came from a single tertiary hospital in low- and middle-income country thus the results may not be transportable to other settings.

## Conclusions

Our study shows that there are more non preventable reasons for readmission but there is a significant number of preventable reasons. Of those, having health-care related infections is the most common that can be addressed in order to avoid readmissions, improve quality of life and reduce hospital expenditures. This study also shows that type of readmission (emergency), and number of medications per day (5–10) are associated with increased likelihood of preventable readmissions.

Overall, this study helped us investigate the possible reasons for readmissions which could be addressed at our level as physicians and by the hospital institution itself. There are more unavoidable circumstances leading to readmissions but there’s also a significant portion of avoidable readmissions that needs to be addressed. Our study helps to provide initial information regarding the measures of hospital readmissions in our country. Acknowledging the evidence can allow hospitals and policymakers to implement quality improvement practices that can reduce preventable readmissions and reduce hospital and healthcare costs. This can also provide an avenue to improve communications between healthcare teams and create programs that encourage better patient discharge preparations and decision making.

The study was done in the hope to address the identified preventable factors for readmissions in a tertiary university hospital in the Philippines. We recommend proper discharge planning and conduct of research such as quality improvement projects for monitoring and trials on effective strategies to reduce these preventable admissions. Moreover, this topic should be further pursued to further identify and implement interventions that can help improve hospital’s performance.

## Supplementary Information

**Additional file 1.** 

**Additional file 2.** 

**Additional file 3.**

## Data Availability

All data generated and analyzed in this study are included in this published article.
