# Supplementary material for: Predictors of readmission in a medical department of a tertiary university hospital in the Philippines
Source: BMC Health Serv Res. 2023 Jun 12;23:617. doi: 10.1186/s12913-023-09608-z (PMC10258940; doi:10.1186/s12913-023-09608-z)
Supplement: Supplementary file 1 — Additional file 1. [file 12913_2023_9608_MOESM1_ESM.docx]

**Supplementary Table 1**

| **Table 1. Readmission Category** | |
| --- | --- |
|  | **n (%)** |
| Planned related to index admission | 72 (22.2) |
| Planned unrelated to index admission | 3 (0.9) |
| Unplanned related to index admission | 221 (68.2) |
| Unplanned unrelated to index admission | 28 (8.6) |
