# Supplementary material for: Predictors of readmission in a medical department of a tertiary university hospital in the Philippines
Source: BMC Health Serv Res. 2023 Jun 12;23:617. doi: 10.1186/s12913-023-09608-z (PMC10258940; doi:10.1186/s12913-023-09608-z)
Supplement: Supplementary file 2 — Additional file 2. [file 12913_2023_9608_MOESM2_ESM.docx]

**Supplementary Table 2**

| **Table 2. Significant correlates of preventable readmission** | | |
| --- | --- | --- |
| **Variable** | **Adjusted odds ratio**  **(95% Confidence Interval)** | **p-value** |
| Emergency vs. scheduled readmission | 3.37 (1.72-6.60) | <0.001 |
| Number of medications per day (5-10 vs <5) | 1.78 (1.10-2.87) | 0.018 |
| With nosocomial infection | 1.86 (1.09-3.17) | 0.024 |
