# Supplementary material for: Predictors of readmission in a medical department of a tertiary university hospital in the Philippines
Source: BMC Health Serv Res. 2023 Jun 12;23:617. doi: 10.1186/s12913-023-09608-z (PMC10258940; doi:10.1186/s12913-023-09608-z)
Supplement: Supplementary file 3 — Additional file 3. [file 12913_2023_9608_MOESM3_ESM.docx]

**Supplementary Table 3**

| **Table 3. Reasons for Readmission** | | | | | |
| --- | --- | --- | --- | --- | --- |
| **Preventable (n=140)** | | | **Nonpreventable (n=184)** | | |
|  | **n** | **%** |  | **n** | **%** |
| Health-care related infection | 60 | 42.9 | Planned readmission | 72 | 39.1 |
| Nonadherence to treatment allegedly due to lack of information | 28 | 20 | Unavoidable progression of disease | 45 | 24.5 |
| Procedure not performed during previous admission | 18 | 12.9 | Process not related to previous episodes | 28 | 15.2 |
| Lack of diagnosis during previous admission | 13 | 9.3 | Unavoidable recurrence of disease | 15 | 8.2 |
| Lack of appropriate alternative centres for delivering the care required (eg.palliative) | 7 | 5 | Acute exacerbation of concomitant process | 15 | 8.2 |
| Inadequate use of drugs (includes inadequate dosage and interactions | 6 | 4.5 | Nonadherence to therapeutic recommendation attributable to the patient | 9 | 4.9 |
| Complication of surgical procedure except healthcare-related infection | 4 | 2.9 |  |  |  |
| Complication of diagnostic test | 4 | 2.9 |  |  |  |
